# The role of self-representation in emotional contagion

**DOI:** 10.3389/fnhum.2024.1361368

**Published:** 2024-05-09

**Authors:** Dan Wang, Changhong Liu, Wenfeng Chen

**Affiliations:** ^1^Department of Psychology, Renmin University of China, Beijing, China; ^2^Department of Psychology, Bournemouth University, Dorset, United Kingdom

**Keywords:** emotional contagion, self-representation, social context, self-concept, interpersonal relationship

## Abstract

Although prior research has implied that emotional contagion occurs automatically and unconsciously, convincing evidence suggests that it is significantly influenced by individuals’ perceptions of their relationships with others or with collectives within specific social contexts. This implies a role for self-representation in the process. The present study aimed to offer a novel explanation of the interplay between social contexts and emotional contagion, focusing on the contextualized nature of self-representation and exploring the social factors that shape emotional contagion. It further posits a causal loop among social contexts, self-representation, and emotional contagion. Drawing from the lens of self-representation, this study concludes with a discussion on potential research directions in this field, commencing with an exploration of the antecedents and consequences of emotional contagion and self-representation.

## Introduction

Emotional contagion, a phenomenon where our emotions are unconsciously influenced by those of our social surroundings (Hatfield et al., 1993), particularly those closest to us, serves as a vital catalyst for social cohesion. This natural process facilitates the rapid transmission of social signals and is innate, evident even in infancy, as infants often respond by crying to the sounds of other crying babies (Herrando and Constantinides, 2021; Salvadori et al., 2021). Emotional contagion is characterized by affective synchrony, manifesting in various levels of synchrony in emotional experience, expression (such as facial and postural expression), and neural and physiological processes (Hatfield et al., 2009). When two individuals’ emotions are dynamically aligned in both form and timing, we refer to this state as affective synchrony, a good indicator of emotional connection and understanding (Wood et al., 2021).

Emotional contagion and empathy share a core feature: a shared emotional experience. However, empathy is a more comprehensive concept that extends beyond emotional contagion. Despite their similarity in shared emotional experiences, they differ in their underlying mechanisms. Empathy comprises two distinct systems: affective empathy and cognitive empathy. Like emotional contagion, the former refers to the automatic emotional response evoked by observing another person’s emotional state (Heyes, 2018). The latter, on the other hand, involves a more intricate process of cognitive control (Isern-Mas and Gomila, 2019). Hatfield et al. (2009) emphasized one aspect of empathy as the ability of people to “feel themselves into” another’s emotions via emotional contagion. According to Hatfield et al. (2009), the primary distinction between empathy and emotional contagion lies in the element of self-other distinction. Empathy involves a clear distinction between oneself and others, whereas emotional contagion operates at a subconscious level, without such discrimination. Instead, it relies on a form of “total identification” where the feelings of the self and others overlap, reflecting an innate ability to resonate with the emotions of others (Decety and Moriguchi, 2007; Håkansson Eklund and Summer Meranius, 2021).

The mirror neuron system (MNS) serves as a potential neural foundation for emotional contagion, bridging the gap between perception and action (Likowski et al., 2012; Paz et al., 2022). Although emotional contagion appears to occur automatically, it is not a purely bottom-up process or reflexive imitation. Several studies suggest that the process of emotional contagion is modulated by various social contextual factors such as relationship intimacy (Kimura et al., 2008; Wróbel, 2018; Lin et al., 2024), social similarity (Stockert, 1994; Paukert et al., 2008), and group identity (Joby and Umemuro, 2022). These results demonstrate that the social connection between interacting partners is a prerequisite for emotional contagion. That is, emotional contagion is more likely to occur in an affiliative social context but is attenuated or absent for those reluctant to interact (Hatfield et al., 2014; Hess, 2021). Thus, emotional contagion is a special emotional reaction of the “self” to the emotions of others (Isern-Mas and Gomila, 2019), a process involving the integration of self-representation and other representation in the social context.

According to embodied simulation theory, individuals simulate others’ emotions through the activation of shared neural and physiological representations between themselves and others, which mirror the others’ emotions, leading to vicarious emotional experience (Gallese, 2006). In essence, the effect of social context on emotional contagion is based on how individuals perceive their relationships with others. This perception is closely linked to their self-representation (Cross et al., 2011). Self-representation involves an individual’s self-perception and how they present themselves to the external world (Thagard and Wood, 2015). How people define themselves in relation to others significantly influences their thoughts, emotions, and behaviors, ultimately modulating perception and understanding of others’ emotions in social interactions (Markus and Wurf, 1987; Fischer et al., 2004; Wang et al., 2015).

Therefore, this study emphasizes the pivotal role of self-representation in emotional contagion. It serves not only as a cognitive framework for perceiving and interpreting the emotions of others but also as a modulator of emotional contagion based on the perceived social relationships within a given context. This review integrates this line of research, exploring how self-representation shapes emotional contagion and how it evolves in diverse social settings. Importantly, previous research has primarily focused on self-other relationships as prerequisites for emotional contagion, overlooking the potential for emotional contagion to, in turn, reshape these relationships. We aim to bridge this gap by synthesizing relevant studies and discussing the dynamic interplay between social context, self-representation, and emotional contagion. This interplay not only affects how we perceive and respond to the emotions of others but also how our relationships evolve over time. Future research directions are also outlined, emphasizing the need to further investigate the complex interplay between social context, self-representation, and emotional contagion. By doing so, we can gain a deeper understanding of the psychological mechanisms underlying social interactions and the role of emotional contagion in shaping our social world.

## How are people contagious to others’ emotions?

The Neurocognitive Model of Emotional Contagion underscores the significance of dynamic synchronization activities between two interacting brains in the emergence of emotional contagion. This synchronization arises from the shared neural activities between individuals (Prochazkova and Kret, 2017). Infants, for instance, demonstrate this ability to share emotions through shared representations of their own and others’ behaviors (Herrando and Constantinides, 2021; Salvadori et al., 2021). By mimicking facial expressions, they not only perceive but also empathize with the emotions of those around them (Decety and Sommerville, 2003). In essence, emotional contagion reflects a match between the perceptions of others’ emotions and their feelings, representing a form of shared representation (Preston and Waal, 2002; Teufel et al., 2010).

Self-other shared representation refers to the phenomenon in which individuals share similar representations or models in cognition, emotion, or behavior with others (Decety and Sommerville, 2003). Individuals create shared cognitive frameworks by mapping emotions onto others, leading to shared emotional experiences (Gallese, 2006). Neuroimaging studies have provided compelling evidence for this shared neural representation. For instance, when an individual experiences disgust or pain, the same brain regions are activated as when observing others experiencing these emotions (Wicker et al., 2003; Singer et al., 2004). The shared neural representation, supported by the MNS, bridges the gap between self and others. This enables individuals to comprehend the intentions of others and share their emotional experiences in a manner that goes beyond the self, allowing the “other” to become another “self” (Ferrari and Gallese, 2007).

However, emotional contagion in real life is not a perfect replication of other’s emotional experiences, as each individual’s mental imagery is inevitably colored by their unique life experiences, making it impossible to grasp the exact emotional state of another person entirely. This limitation is a testament to the silent yet significant effect of self-representation on emotional contagion (Arizmendi, 2011). Indeed, the role of self-representation in emotional processing becomes even more evident when considering studies on mental disorders. For instance, individuals with autism spectrum disorders often exhibit abnormalities in brain function activation when recognizing their own faces or attempting to comprehend the emotions of others (Dapretto et al., 2006; Kita et al., 2011). These findings underscore the crucial role of a well-functioning self-representation system in establishing and maintaining emotional connections with others. Moreover, the interdependence between self-representation and emotional contagion becomes apparent.

## Overlapping neural substrates of emotional contagion and self-representation

Humans have the ability to understand and perceive the emotions of others by invoking neural activity or internal simulation associated with their own emotional experiences (Preston and Waal, 2002). This suggests that there may be overlapping neural mechanisms involved in both self-related processing and the processing of others’ emotions. Although self-related processing is multifaceted and encompasses aspects ranging from conceptual to bodily, core brain regions emerge as the nexus of this multifaceted self-concept. Hu et al. (2016) performed activation likelihood estimation (ALE) meta-analyses to investigate this shared neural representation, focusing on the physical and psychological self. They found that the dorsal anterior cingulate gyrus (dACC), left inferior frontal gyrus (IFG), and insula are key regions involved in self-representation.

These regions are also crucially involved in emotional contagion. For instance, some neuroimaging studies revealed that the insula and dACC were activated when individuals observed others’ emotions (Singer et al., 2004; Cheng et al., 2010). Furthermore, compared to strangers, the intensity of activation in these brain regions is greater when perceiving the emotions of a close one, which may imply that self-related stimuli can easily be mapped to one’s representation system. The IFG has been demonstrated to play a crucial role in self-representation (Sugiura et al., 2000; Uddin et al., 2005), and there is also consistent evidence for the involvement of the IFG in emotional contagion (Shamay-Tsoory, 2011). Jabbi et al. (2007) found that observing positive and negative facial expressions activated parts of the IFG, and another study showed that cortical lesions involving the IFG are associated with impaired emotional contagion and deficits in emotion recognition (Shamay-Tsoory et al., 2009).

**Figure 1:**
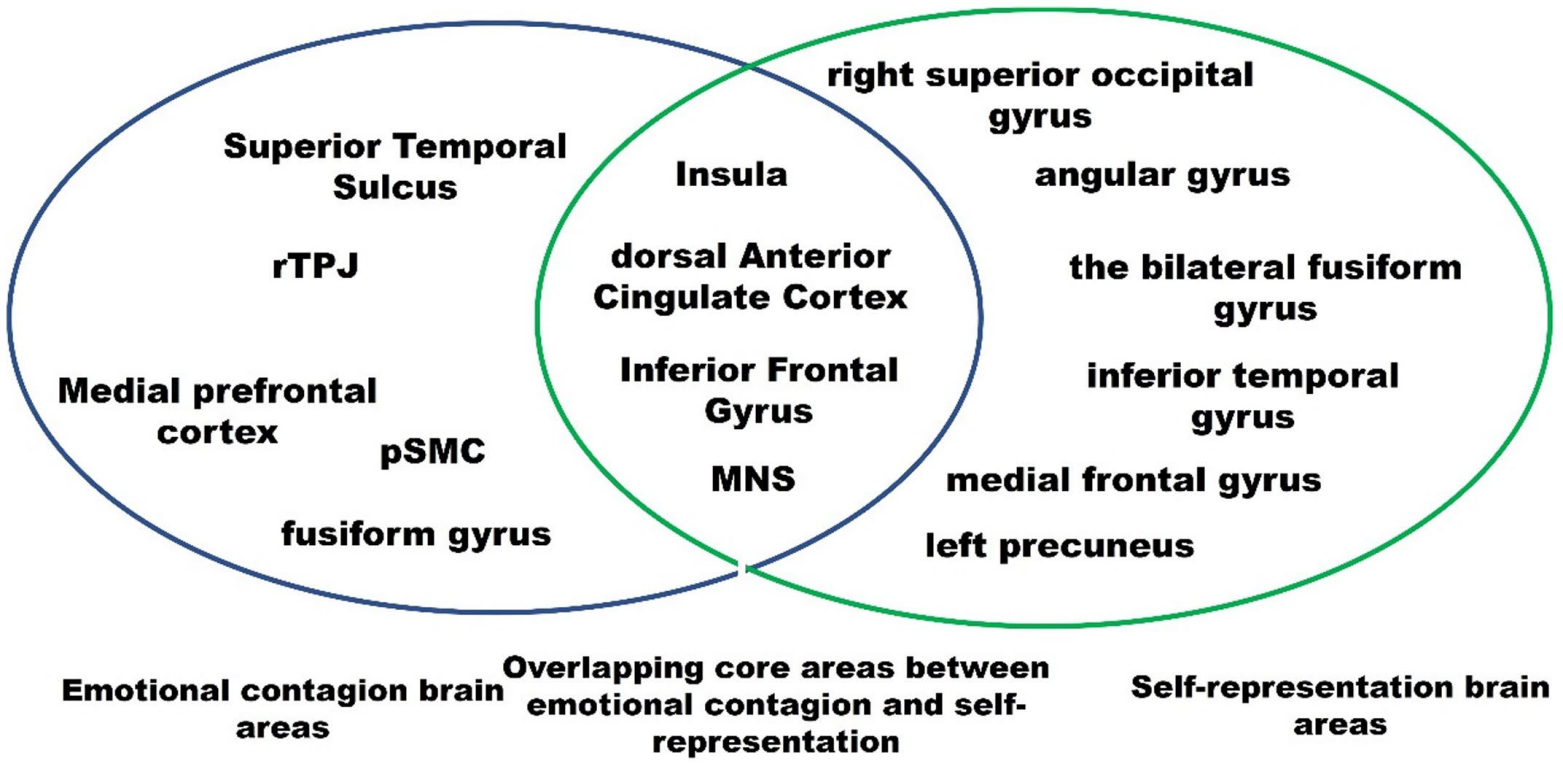
Overlapping core brain regions of emotional contagion and self-representation.

Another overlapping network for emotional contagion and self-representation is the MNS, including the IFG, inferior parietal lobule (IPL), insula, and supplementary motor area (SMA). Molnar-Szakacs and Uddin (2013) argue that understanding self and others belongs to the same system. By prioritizing access to our own physical and mental states, we can then better understand the physical and mental states of others through embodiment and mentalizing, and the MNS and default network both support these cognitive processes (Wu et al., 2015). MNS provides a simulating mechanism for emotional contagion, whereby we understand others’ behavior and emotions by “embodying” them ourselves (Gallese and Sinigaglia, 2011). The observer’s MNS uses a mechanism that resembles an imitation mechanism to process others’ emotions. In this process, other’s emotional states are mapped to the observer’s motor repertoire. If the other person is more similar and familiar to the observer, the mapping mechanism produces a better fit, resulting in increased neural resonance. Figure 1 shows the overlapped brain regions between self-representation and emotional contagion.

## Emotional contagion and self-representation in a social context

### Effect of social context on emotional contagion

Emotional contagion is not merely a replication of feelings; rather, it is a complex phenomenon influenced by a person’s cognition, past experiences, and various social contexts and cues (Hatfield et al., 2014). Interpersonal relationships play a pivotal role in shaping emotional contagion, and the effectiveness of emotional information transmission during social interactions hinges on individuals’ perception of their relationship with others. People are more prone to experiencing emotional resonance with those who share affiliations with them, such as members of their ingroup, partners, or individuals with collaborative intentions. A study conducted by Wróbel (2018) manipulated the closeness of relationships to investigate its impact on emotional contagion. The findings revealed that “second-hand” happiness, where senders watched emotional videotapes and subsequently transmitted their perceived emotions to receivers, occurred exclusively among friends and not among strangers. More recently, Lin et al. (2024) investigated the influence of interpersonal closeness on the intensity of emotional contagion and physiological synchrony between interacting partners. In this study, pairs of friends and strangers participated, with the sender watching a film clip while the observer passively observed the sender’s facial expressions. The results demonstrated that under conditions of positive emotion, more significant emotional contagion and physiological synchrony (in terms of heart rate and heart rate variability) were more likely to occur among friend dyads compared to stranger dyads. Furthermore, relationships can also modulate neural synchronization during emotional interactions. Romantic partners, for instance, exhibit greater behavioral synchronization and brain-to-brain neural synchrony during emotional communication compared to strangers (Kinreich et al., 2017). This underscores the intricate interplay between interpersonal relationships and the dynamics of emotional contagion.

Other factors, such as social power within interpersonal relationships, have also been shown to influence the dynamics of emotional contagion (Kimura et al., 2008). Beyond interpersonal bonds, the impact of social identity, especially in the context of group membership, has been identified as a significant factor in emotional contagion. This was evident by Joby and Umemuro (2022) which reveals that emotional contagion and favorable social attitudes, including trust, empathy, liking, bonding, and prosocial orientation, are notably more prevalent within ingroup interactions compared to out-group interactions. This suggests that the nature and strength of our social bonds and our perception of group membership play a crucial role in shaping our emotional responses and the transmission of emotions within social groups.

### Social context and the self: contextualized self-representation

Self-concept, as described by James (2018), is a multifaceted construct that can be represented in various forms. Sedikides and Brewer (2001) identified three fundamental types of self-representation: the individual self, the relational self, and the collective self. The individual self encapsulates those aspects that distinguish a person from others, highlighting their unique characteristics and identity. In contrast, the relational self emphasizes the similarities between one’s representation of self and others. It incorporates attributes shared with close individuals and defines the roles within dyadic relationships. The collective self, on the other hand, encapsulates an individual’s intergroup aspect. It comprises attributes that are shared with members of the ingroup and differentiated from outgroups, reflecting one’s membership in a particular social group (Brewer and Gardner, 1996; Sedikides et al., 2011). The relational self and the collective self can be collectively referred to as the social self. This aspect of self-representation captures the overlap between one’s representation of self and others (Ellemers et al., 2002). Importantly, these three types of selves coexist, and individuals can switch between perceiving themselves as distinct individuals, relational partners, or interchangeable group members. Therefore, self-representation serves not only for self-awareness but also to represent the self-other relationship and interpersonal interactions (Tsakiris, 2017).

However, the dominance of a particular self-representation depends on an individual’s motivational state or contextual factors (Andersen and Chen, 2002). For instance, when an individual’s group identity is emphasized, the collective self becomes prominent (Turner et al., 1987). Similarly, when we are in the presence of a significant other, memories related to the self and that significant other, both in abstract and experiential forms, are activated, manifesting as the relational self (Hinkley and Andersen, 1996).

### Interaction between social context and emotional contagion: the role of self-representation

Humans are constantly engaged in the construction and reconstruction of their social selves throughout their lifetimes. This process is deeply influenced by social interactions, life experiences, and feedback from others. The self-concept is a dynamic and ever-evolving representation that adapts and changes in response to these diverse inputs (Mead, 1913; Oyserman et al., 2012).

Social contextual cues and individual motivational states play a crucial role in shaping self-representation. For instance, when individuals are immersed in close relationships, the relational self, characterized by a strong preference for interpersonal connection, becomes particularly prominent (Aron et al., 2013). A key aspect of the relational self is the overlap between self and others, which occurs through a process of self-expansion. In this process, individuals integrate resources, and perspectives of other individuals into their self-concept, emphasizing the representational similarities between the self and others (Aron et al., 1991; Zi and He, 2019).

Driven by the motivation for self-expansion, the boundaries between self and others are often redefined, leading to updates in self-representation that reflect the relationship between the self and others. This expansion of the self-concept results in a shared cognitive construction of the self and others, where it becomes difficult to distinguish memories and traits that are relevant to the self from those that are relevant to close others (Mashek et al., 2003).

Furthermore, this expansion facilitates the brain’s ability to represent the perceived emotions of others as if they were the emotions of the self. For instance, in close relationships, people tend to internalize their partner’s positive emotions as their own (Meixner and Herbert, 2018). fMRI studies have provided further evidence that when individuals perceive the emotions of significant others, brain regions associated with self-representation functions are more strongly activated, and the activation pattern is similar to when they experience the emotions themselves (Singer et al., 2004; Cheng et al., 2010). In contrast, non-affiliative relationships (e.g., hostile or competitive relationships) tend to activate the individual or independent self-representation (Cristina-Corina, 2012), resulting in less emotional resonance or even opposite emotional responses (Lanzetta and Englis, 1989; Wróbel and Imbir, 2019).

The influence of self-representation on emotional contagion extends beyond individual interactions to encompass group dynamics. Gardner et al. (2002) found that the activation of the collective self leads individuals to perceive the success of group members as a positive event, while the activation of the individual self may evoke unpleasant feelings in response to such success. Individuals with a strong sense of belonging to a group tend to merge their personal identity with that of the group, resulting in a blurred boundary between the individual self and the collective self (Swann et al., 2012). This process reflects a shift from an emphasis on the individual self to an emphasis on group identity within the self-concept. According to social identity theory (Hogg, 2016), people derive a sense of self-esteem and identity from their membership in social groups, and a highly integrated self is characterized by a strong identification with the group and prioritization of group identity over personal identity (Liu et al., 2022). Therefore, when the collective self dominates, people are more likely to understand and view the world based on group members’ perspectives, accept the group’s views and emotions, and value the connection with the group (Hareli and Rafaeli, 2008; Blocker and McIntosh, 2017; Han, 2018).

Emotional contagion is not only a natural outcome of social interactions but also an antecedent that can profoundly shape interpersonal relationships and social behavior. It occurs when individuals unconsciously catch and reflect the emotions of those around them, often leading to a shared emotional experience. This process not only strengthens social bonds but also alters one’s perception of self and others. Indeed, emotional mimicry, a common behavior associated with emotional contagion, involves unconsciously mirroring the facial expressions and gestures of others. Studies have demonstrated that this mimicry enhances feelings of affiliation and closeness between interaction partners (Cheung et al., 2015; Hess et al., 2016). Those who engage in emotional mimicry tend to develop a self-concept that is more interdependent, emphasizing the importance of interpersonal relationships and the prioritization of others’ emotions and needs (Chartrand and Bargh, 1999). Even mere action imitation can alter interactants’ self-concept, with the mimicked individual’s self-concept becoming more interdependent and the imitator experiencing enhanced feelings of interdependency (Ashton–James et al., 2007; Hamilton, 2017). Furthermore, emotional contagion goes beyond mere mimicry. It involves a deeper level of self-involvement, where individuals share feelings with others, even for brief moments, strengthening their emotional connection (Mariadhas, 2019; Mayo et al., 2023). This shared emotional experience can have great effects on individuals’ sense of self and their relationships with others.

Another notable aspect of emotional contagion is its ability to induce synchrony in attention, emotion, and behavior. When people are emotionally synchronized, they are more likely to perceive themselves as part of a larger group or collective, blurring the boundaries of independence (Good et al., 2017). This affective synchrony enhances not only emotional integration but also perceptual coherence, bridging the psychological distance between individuals and fostering a sense of “we” rather than “you” and “I.”

**Figure 2:**
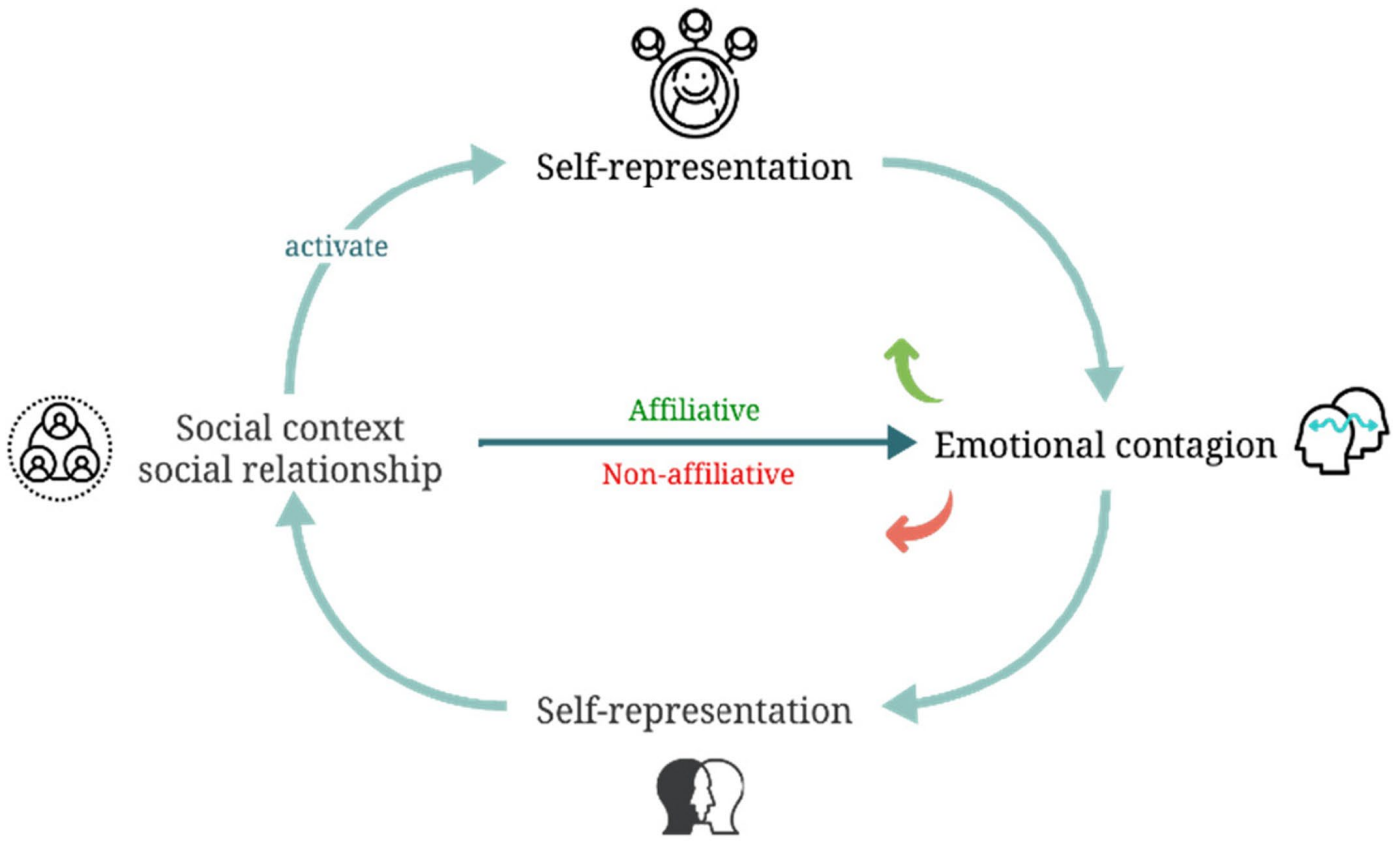
Schematic representation of the interplay between social context, self-representation, and emotional contagion.

Overall, emotional contagion is a process that is complicated and linked to self-experience. The way individuals respond to the emotions of others is significantly influenced by their perception of their relationships, which involves alterations in their self-representation. Social contexts play a pivotal role in regulating emotional contagion by shaping an individual’s self-representation. Specifically, self-representation is dynamically constructed and activated during interpersonal interactions, contingent on the prevailing social contexts. This, in turn, affects their emotional perception, cognitive functions, and information processing, ultimately either enhancing or weakening their capacity to perceive and comprehend the emotions of others and exhibiting adaptive emotional responses. Conversely, emotional synchrony facilitates connection and mutual understanding between individuals, shaping how they view themselves and others. This shift strengthens emotional bonds among individuals, thereby influencing their social behavior (see Figure 2).

## Conclusion and future directions

This study integrates theories and empirical research from self-concept and emotional contagion to propose a mechanism that explains the interaction between social context and emotional contagion, emphasizing the crucial role of self-representation. Drawing inspiration from embodied simulation theory, we posit that the capacity to share in others’ emotions is also rooted, at least in part, in self-representation. When an individual perceives the emotions and actions of others, internal self-representations associated with these experiences are activated, as if the observer were experiencing them directly (Gallese, 2006). Moreover, a significant and novel aspect of our proposal lies in its articulation of the dynamic interplay between emotional contagion and social context. The social context shapes individuals’ emotional responses to the emotions of others by activating specific self-representations. In short, when individuals are situated within a particular social context, they may become aware of their identity, roles, or relationships within that setting, and contextualized self-representation is activated. Consequently, their emotional responses and behaviors are influenced by these activated self-representations. Additionally, the downstream effects of emotional contagion manifest in the enhancement of interdependent self-representation, which in turn fosters social connection. This underscores the intricate link between emotional contagion, self-representation, and social context, highlighting the dynamic and interactive nature of these processes.

Based on the dynamic interplay between social contexts, self-representation, and emotional contagion, several future research directions are proposed. First, while we have established the relationship between these constructs through empirical research and theory, there is still a need for direct evidence validating this model. Future studies can explore how self-representation shapes emotional contagion in social interactions. For instance, it would be interesting to investigate whether specific social contexts trigger different forms of self-representation, such as a relational or individual self, and how these forms predict an individual’s susceptibility to emotional contagion from others.

Second, longitudinal studies could be conducted to assess the evolution of self-expansion and its impact on emotional contagion across various stages of interpersonal relationships. Such studies would provide valuable insights into the dynamic and interactive nature of these processes, allowing us to better understand how changes in self-representation affect emotional contagion over time.

Third, although previous research has demonstrated the top-down modulating effects of social relationships on emotional contagion (Kimura et al., 2008; Wróbel, 2018; Franklin, 2019), there is a need to further explore the reverse relationship. Few studies have examined how emotional contagion influences social relationships and other prosocial behaviors. Future research should aim to investigate the bidirectional nature of this relationship and explore whether contextualized self-representation plays a role in mediating these effects.

Fourth, while much of the existing research has focused on interpersonal emotional contagion, it is important to recognize that emotional contagion can have significant effects on intergroup and ingroup behaviors in organizations. Future research should explore the extent to which emotional contagion influences intergroup dynamics, such as group cohesion, cooperation, and conflict resolution. This line of study has the potential to yield important insights into how emotional contagion can shape organizational behavior and performance.

Finally, it would be interesting to explore the role of cultural factors in shaping the relationship between social context, self-representation, and emotional contagion. Different cultures may have distinct norms and values that influence how individuals perceive themselves and others, which could, in turn, impact the extent of emotional contagion within those cultures.

In summary, these future research directions offer opportunities to further understand the complicated relationships between social contexts, self-representation, and emotional contagion. By addressing these gaps, we can gain deeper insights into the mechanisms underlying emotional contagion and its impact on social interactions and relationships.

## Data availability statement

The original contributions presented in the study are included in the article/supplementary material, further inquiries can be directed to the corresponding author.

## Author contributions

DW: Conceptualization, Writing – original draft, Writing – review & editing. CL: Writing – review & editing. WC: Writing – review & editing, Conceptualization, Funding acquisition, Supervision.
